# Has COVID-19 Changed China's Digital Trade?—Implications for Health Economics

**DOI:** 10.3389/fpubh.2022.831549

**Published:** 2022-03-02

**Authors:** Feng Hu, Liping Qiu, Xun Xi, Haiyan Zhou, Tianyu Hu, Ning Su, Haitao Zhou, Xiaolei Li, Shaobo Yang, Zhigang Duan, Zenan Dong, Zongjian Wu, Haibo Zhou, Ming Zeng, Ting Wan, Shaobin Wei

**Affiliations:** ^1^Global Value Chain Research Center, Zhejiang Gongshang University, Hangzhou, China; ^2^School of Management, Shandong Technology and Business University, Yantai, China; ^3^Institute of Spatial Planning and Design, Zhejiang University City College, Hangzhou, China; ^4^School of Information Engineering, Zhengzhou University, Zhengzhou, China; ^5^School of MBA, Zhejiang Gongshang University, Hangzhou, China; ^6^China State Construction International Investments (Zhejiang Province) Ltd., Hangzhou, China; ^7^Research Centre, Shanghai Yice Think Tank, Shanghai, China; ^8^Jinhua JG Tools Manufacturing Co., Ltd., Jinhua, China; ^9^Sales Department, Estone SRL, Carrara, Italy

**Keywords:** COVID-19, digital trade, Chinese exports, natural experiments, generalized difference-in-differences

## Abstract

Digital technologies have played a significant role in the defense against the COVID-19 pandemic. This development raises the question of whether digital technologies have helped Chinese exports recover quickly and even grow. To answer this question, we study monthly data on Chinese exports to 40 countries/regions from January 2019 to June 2020 and covering 97 product categories. The study takes the COVID-19 outbreak as a natural experiment and treats digital trade products as the treatment group. Using a generalized difference-in-differences (DID) approach, we empirically investigate how this major global public health crisis and digital trade have influenced Chinese exports. Our empirical analysis reveals that the COVID-19 pandemic has inhibited China's export trade overall, digital trade has significantly promoted trade, and the supply mechanism has played a significant role in promoting the recovery of exports. Heterogeneity tests on destination countries/regions reveal that digital trade has significantly promoted exports to countries/regions with different income levels, with a more significant effect on low-risk destinations than on high-risk destinations. The sector heterogeneity test demonstrates that digital trade has enhanced the export recovery of sectors dealing in necessities for pandemic prevention. Other robustness tests, including parallel trend and placebo tests, support the above conclusions. Finally, we extend the research conclusions and discuss their implication for health economics and the practice of fighting COVID-19.

## Introduction

COVID-19 has become one of the most severe global public health crises and has led to a serious economic crisis. It has slowed economic growth and worsened employment prospects (1), and it may continue to impact the world (2). In almost all major countries around the world, 2020 saw a downturn in GDP, although China still achieved GDP growth of 2.3%, which is lower than before the outbreak of the pandemic. Notably, once the pandemic broke out in Wuhan, China, in early 2020, the Chinese government ordered an immediate lockdown in Hubei Province and restricted industrial activities after the Chinese New Year holiday. Although the lockdown measure is thought to have devastated the national economy and international trade, the loosening of the regulation may have risked a recurrence of the pandemic (3). According to the National Bureau of Statistics of China, China's GDP growth rate changed from negative to positive in Q2 2020, as did the growth rate of cargo imports and exports in Q3 2020, despite the shocks caused by the pandemic. These outcomes prove that the impact of a general lockdown on the economy is temporary and controllable and does not alter megatrends of economic development (4).

Given the economic recession caused by the general lockdown, the capacity for economic recovery capacity after the lifting of the lockdown is particularly important. We may recall that the lockdown implemented in the early stage of the pandemic forced people to isolate themselves at home and to work from home, and the combination of proactive prevention measures and smart technical solutions minimized the transmission of COVID-19 in some countries, e.g., China and South Korea (5). Examples of popular smartphone applications include the Health QR Code and Journey QR Code, which the Chinese government rolled out to identify potential transmission routes and to safeguard economic recovery based on big data on population movement, although certain countries have been concerned about consumer privacy and legal rights issues arising from the collection of positioning information. After the lockdown was lifted, the Chinese government implemented information technology (IT)-based initiatives to stimulate consumption and thus drive economic recovery. For example, local governments issued electronic consumption coupons in cooperation with China's prevailing e-pay platforms, such as UnionPay Quickpass, Alipay, and WeChat. However, it has been pointed out that the digital divide further expanded during the pandemic (6). It is undeniable that digital technologies have become one of the safeguard measures for fighting the pandemic and restoring economic activity.

On the world market, the pandemic has forced consumers to move from offline to online consumption. The demand for e-commerce, which is based on Internet platforms, has grown abruptly all over the world (7, 8). One major reason is that digital trade, which operates on cross-border platforms, can help a company quickly identify new partners once its supply chain is interrupted. In particular, the quick matching of pandemic prevention materials between suppliers and buyers across the world has helped alleviate the impact of the pandemic on world trade and accelerate the recovery of global trade chains.

This paper aims to examine the role of digital technology in promoting China's export recovery and growth as COVID-19 has severely suppressed international trade. in particular, this paper takes the pandemic as a natural experiment under a seminatural experimental approach to policy effect assessment. The pandemic has severely affected all forms of trade, but different forms of trade may suffer different degrees of damage, and the feedback may also differ. We assume that some digital trade performs have been better than others during and after the pandemic, and we treat them as the treatment group. Conventional forms of trade are treated as the control group. To review how the treatment and control groups influence Chinese exports in the pandemic, we establish a generalized difference-in-differences (DID) model through the interaction of grouping and month dummy variables to assess the unique impact of digital trade during the pandemic. The studied data are the gross values of 97 product categories falling under the China Customs Sections and Divisions that China exports to 40 major countries/regions 1 year ahead of the pandemic and half a year thereafter.

This study is potentially significant in three ways. First, taking the pandemic as a natural experiment can reveal the unique impact of digital trade on Chinese exports, thereby providing empirical evidence to facilitate Chinese exports for self-initiated transformation and to respond to emergencies. Second, this study empirically tests the influence of digital trade on Chinese exports. The experience gathered from Chinese practices may provide a reference for the recovery of the global economy and trade. Third, the pandemic has seriously endangered public health and caused an enormous impact on economic growth. This article further proposes implications for health economics to promote the long-term healthy development of the world economy.

The subsequent contents are organized as follows. Section Theoretical Basis and Research Hypotheses proposes the hypotheses of this paper based on a review of the theoretical background and the performance of digital technologies in foreign trade and the defense against the COVID-19 pandemic. Section Research Methods introduces the empirical models and data. Section Regression Analysis performs empirical analysis and tests of the models. Finally, section Conclusion and Discussion discusses conclusions, implications, and limitations of this study and suggestions for future research.

## Theoretical Basis and Research Hypotheses

### Digital Trade and China's Practices

The development of conventional international trade benefits from large-capacity transport vehicles emerging from three industrial revolutions. The easy availability and wide use of computer networks have led to Internet-based platforms, including cross-border e-commerce (CBEC) platforms. In the 4th industrial revolution, highly digital technologies such as big data and blockchain have promoted the rapid digital transformation of traditional international trade. Therefore, digital trade will become the leading pattern of international and domestic trade in the coming years (9). Digital trade was initially defined as the trade of digital products and services, excluding physical products (even products with digital features) (10, 11). Later, the definition was expanded to stress that digital trade is the trade realized by digital means and that it includes conventional international trade that involves the use of Internet technologies, i.e., Internet transactions of physical and digital products and services in physical or digital forms (1, 12). In general, digital trade is characterized by modern information networks as carriers, online dealing supported by knowledge and information digitalization technologies, and the transfer of physical and digital products and services.

China's digital transformation of trade originates from the application of the “Internet plus” in the fields of both conventional trade and e-commerce^1^, giving birth to the new pattern of Internet-based CBEC, which has developed quickly and been acclaimed as the “new engine of China's foreign trade” (13, 14). Internet-based CBEC significantly reduces the intermediate trading steps (1), effectively decreases matching costs, and improves trading efficiency (15, 16). At the same time, digital transformation has altered the process of consumer value creation and cocreation (17). Furthermore, as digital technologies are applied and developed, CBEC is endowed with more features in more dimensions, and China's CBEC will ultimately iterate into digital trade (18). China's leading Internet companies are seeking breakthrough innovations by launching digital innovative strategies such as the “Digitalize Global Trade” strategy of Alibaba.com. Furthermore, China's national strategies are oriented toward digital transformation in the Industry 4.0 era through the implementation of strategies such as intelligent manufacturing and Made in China 2025.

### COVID-19 Pandemic, Foreign Trade, and the Application of Digital Technologies

The outbreak of the COVID-19 pandemic has brought exponential growth in the use of information and communication technologies (ICTs) (19) to mitigate the unfavorable impact of the physical distance resulting from the lockdown and isolation. In some countries, advanced digital technologies have been applied for pandemic surveillance, control and analysis, thus feeding decision makers with more precise information more quickly and ultimately driving the recovery of economic activity (20, 21). The COVID-19 crisis has accelerated technological innovation and integration, promoting the digitalization-based strategic transformation of the global economy (22). This development highlights that digital transformation is crucial for mitigating the economic recession, maintaining well-being, and accelerating economic recovery (1).

Digital trade is a new form of trade established based on the new generation of ITs such as big data, cloud computing, the Internet of Things, and artificial intelligence, and it has new features that are different from those of conventional forms of trade. Digital trade utilizes ICTs to realize the efficient exchange of physical goods, digital products and services, and digital knowledge and information. It overcomes the damage to the global economic ecosystem caused by pandemic containment measures implemented in various countries. In particular, it alleviates the spatial limitation of labor flow. For this reason, digital trade could account for as much as 62.8% of the total trade volume worldwide as global trade suffered tough shocks in 2020. Digital trade is promoting a profound revolution of global value and innovation chains and has become a key driving force for global trade recovery in the post-pandemic period.

Based on the above analysis, we believe that digital technologies enable digital trade to substitute for face-to-face trade in the pandemic context. Digital trade, including CBEC, digital media and communication services, grew quickly and promoted the development of innovative forms of global trade and stable growth in the global trade volume. Therefore, we propose the following hypothesis:

**Hypothesis 1. Digital trade is positively related to exports**.

How have digital technologies played an important role in the fight against the pandemic and helped boost the recovery and development of global trade? The collapse of global trade triggered by the COVID-19 pandemic occurred due to mandatory preventive measures. Baldwin and Tomiura (23) pointed out that COVID-19 has impacted global trade from both the demand and supply sides and has been accompanied by increased supply chain contagion in the trade of intermediate goods. This paper attempts to explore how digital technologies drive global trade recovery from the demand and supply perspectives.

From the supply (exporter) perspective, the physical distance maintained by measures such as social distancing has effectively curbed the spread of the pandemic but simultaneously shrunk the labor market. The suspension of production, e.g., the shutdown of factories and the interruption of supply chains, has reduced the scale of production, thereby decreasing the supply of exports. On the supply side, digital technologies can smooth the way for export trade through the short-term use of Internet channels. Products and services supplied through the use of digital technologies are included within the scope of digital trade. First, online interviews and employment are revitalizing the labor market. Second, working from home and online conferences arising in response to pandemic prevention are sustaining business operations. Third, digital trade platforms are offering cost-effective and efficient trade solutions. For instance, online exhibitions reduce matching costs and time compared to physical exhibitions. Additionally, through CBEC service platforms, manufacturers that have stopped production due to supply chain disruptions can quickly identify new suppliers and establish possible partnerships. On the basis of the above analysis, we propose the following hypothesis:

**Hypothesis 2. Digital trade utilizes digital services to reduce trading costs and accelerate export recovery**.

From the demand (importer) perspective, demand countries suffering seriously from the pandemic may implement lockdowns, causing high unemployment and a loss of labor income, which directly affects total demand (24). In addition, fear of the pandemic has prevented members of the general public from leaving their homes, and the shrinkage of retail shops has also reduced total demand. During the pandemic, digital trade has substantially promoted trade recovery by activating non-contact demand, and Internet-based retailing has substituted for shrinking physical shops. Meanwhile, insufficient domestic supply has caused a significant increase in cross-border online orders, which in turn has stimulated export enterprises on the supply side to maintain business operations. The transition of demand from offline to online will continue and may have profound effects on future global trade. Based on the above analysis, we propose the following hypothesis:

**Hypothesis 3. Digital trade provokes partner countries' demand potential and thus promotes a quick recovery of exports**.

## Research Methods

### Empirical Models

DID models have been widely applied in studies on policy effects, and they are believed to be an effective approach to differentiating time trends and policy effects and an effective empirical method to solve endogeneity problems in economic and financial research (25). In general, the design of a DID model is based on identifying a treatment group, which is subject to government policy interference, and a control group, which is free from such interference. However, in the real world, some policies apply to all individuals, such as the abolition of the elite recruitment system (26), making it hard to identify a control group that is completely free from undesired interference. Nevertheless, by identifying the systematic difference that a policy change impact may have on a certain dimension, it is possible to construct treatment and control groups by dividing the samples by the degree of systematic influence (27–29).

We attempt to apply this idea to our study on the impact of the COVID-19 pandemic on Chinese exports. First, nobody could forecast the COVID-19 pandemic until it broke out. Therefore, it can be regarded as a pure exogenous shock, avoiding the endogenous problem of the shock itself. Second, the pandemic had an impact on all countries worldwide in a short period of time, and Chinese exports of goods were also affected. Considering that digital trade has played a significant role in fighting the pandemic (1), we divide Chinese exports based on the degree of digitalization of products to acquire the control and treatment groups, thereby observing how digital products have influenced Chinese exports before and after the pandemic.

After the pandemic broke out, the change in the volume of Chinese exports to major destination countries may result from (1) the time-dependent effect and (2) the pandemic-dependent effect. Through this approach, we established a DID model that is based on the universal application of pandemic prevention measures.


(1) $$\begin{array}{l} \begin{array}{ccc} LnEXP_{itc} & = & \alpha_{0}+\alpha_{1}CBEC_{i}+\alpha_{2}Covid19_{t} \end{array}\\ \;\begin{array}{c} +\alpha_{3}CBEC_{i}*Covid19_{t}+\alpha_{4}X_{tc}+\varepsilon_{itc} \end{array} \end{array}$$

where *i* stands for Harmonized Commodity Description and Coding System (HS) 2-digit products; *t* stands for the month; and *c* stands for the major destination countries/regions of exports. The explained variable *LnEXP*_*itc*_ stands for the logarithmic value of the trade volume of product *i* exported from China to country *c* in month *t*. The explanatory variables include the individual differential variable *CBEC*_*i*_, which indicates whether product *i* is a digitalized product, and the time differential variable *Covid*19_*t*_, which indicates whether month *t* is subject to the impact of COVID-19. The coefficient of the interaction term *CBEC*_i_*Covid19_*t*_ measures the impact of the pandemic on product exports and is the core coefficient that our study focuses on. *X*_*tc*_ is the control variable of country *c* in month *t*. ε_*itc*_ stands for other random disturbance terms that affect product exports.

We further use a two-way fixed effects model, which is a standard panel data model, to separately control for individual and time fixed effects and mitigate omitted variable bias. We add month, product, and sector fixed effects to Model (1) and eliminate the individual and time differential variables to avoid strict multicollinearity. The model is thus modified as follows:


(2) $$\begin{array}{l} \begin{array}{ccc} LnEXP_{itc} & = & \alpha_{0}+\alpha_{1}CBEC_{i}*Covid19_{t}+\alpha_{2}X_{tc}+v_{t} \end{array}\\ \;\begin{array}{c} +u_{i}+\omega_{it}+\varepsilon_{itc} \end{array} \end{array}$$

where *i* = 1, 2, …76, 78, …98 and *t* = 2019−01~2020−06.

Lastly, to analyze the mechanism through which digital trade promotes export recovery, we add country/region-characteristic variable *H*, which separately characterizes the action mechanism of demand and supply. The mechanism testing model is as follows:


(3) $$\begin{array}{l} LnEXP_{itc}=\alpha_{0}+\alpha_{1}CBEC_{i}*Covid19_{t}*H\\ \;+\alpha_{2}CBEC_{i}*Covid19_{t}+\alpha_{3}X_{tc}+v_{t}+u_{i}\\ \;\begin{array}{c} +\omega_{it}+\varepsilon_{itc} \end{array} \end{array}$$

### Variables and Sources

#### Explained Variable

The explained variable **LnEXP**_**itc**_ stands for the logarithmic value of the trade volume of HS 2-digit products exported from China to major countries/regions. It measures the level of export trade. The 43 export destination countries/regions are sourced from the Monthly Statistics Bulletin of the General Administration of Customs of the People's Republic of China. The export trade volume covers 97 product categories included in the China Customs Sections and Divisions^2^. Considering that China Customs increased Division 99 (*articles of B2B cross-border e-commerce in simplified customs procedures*) in July 2020, we selected a time range of 18 months from January 2019 to June 2020 to avoid a data shortage due to the change in statistical coverage. The data are sourced from the China Stock Market and Accounting Research (CSMAR) database. Exports from China to major countries/regions in December 2019 are corrected based on the China Customs database. In the empirical analysis, the export volume data are transformed into natural logarithms after adding 1 so that zero trade flows are not eliminated.

#### Core Explanatory Variables

Individual differential variable CBEC_i_. As analyzed above, products sold based on CBEC platforms have digital characteristics. Following the method of Ma et al. (18) for identifying products traded on CBEC platforms, we identified 1,413 HS 8-digit^3^ CBEC products. Notably, natural shock experiments are subject to the impact of objective conditions and data acquisition. When monthly data were considered to increase the time series, we could only obtain the monthly export data published by China Customs on products under the HS 2-digit Section and Division system. To align the product category levels, we first convert the HS 8-digit products into international standard-compliant HS 6-digit products and thus identify 1,013 product varieties, although such practice involves information omission due to the reduction in the number of individual products. Then, we count the number of HS 6-digit products under the HS 2-digit system to calculate the percentage of products traded through CBEC channels. Using this percentage, we rate the trade into five levels of digitalization, namely, “fully digitalized,” “highly digitalized,” “moderately digitalized,” “minimally digitalized,” and “not at all digitalized.” Furthermore, with reference to Moser and Voena (30), a product category is included in the treatment group as long as one product of that category is sold on CBEC platforms. Moreover, we will examine the dosage effect later in this paper by reconstructing the control and treatment groups through different combinations of digitization levels (e.g., by regarding full and high digitization levels as the treatment group and the other two levels as the control group) to help determine the robustness of the identification strategy.

Time dummy variable Covid19_t_. The time of the outbreak of the COVID-19 pandemic in Wuhan, China, i.e., January 2020, is held as the shock point of the natural experiment. A value of *Covid*19_*t*_ = 0 is assigned for the period before the outbreak of the pandemic, and a value of *Covid*19_*t*_ = 1 is assigned for the period thereafter.

Interaction term CBEC_i_*Covid19_t_. The coefficient of the interaction term is meaningful only after the outbreak of the pandemic and only for the experimental group. In other words, it measures the impact of digital trade on exports after the outbreak of the pandemic.

#### Control Variables

The trade gravity model is among the most popular models in global trade research. We incorporate the common assumption of the gravity model into our control variables. The classical gravity model assumes that the trade volume positively correlates with the economic scale of trading countries and negatively correlates with the distance between them (31).

*relat*_*gdp*_*tc*_. GDP is typically used to measure the market economy scale of a country. This measure can reflect the fluctuation in demand in the global market during the COVID-19 pandemic. This study uses the ratio of the GDP of export destination countries/regions to China's GDP in the same period. Considering that most countries/regions publish quarterly and yearly GDP data, we perform quadratic linear interpolation on quarterly GDP data to obtain monthly estimates following Kisman (32). The data are sourced from the CEIC database.

*lndis*_*cap*_*c*_. The logarithm of the geographical distance between the capital of China and that of an export destination country/region is used to measure the distance between China and that country/region. The data are sourced from the GeoDist database of the French Institute for Research in the Field of International Economics (CEPII).

The trade gravity model has been continuously expanded in subsequent studies by introducing exogenous variables. Frankel et al. (33) considered the effects of cultural and geographical factors, assuming that the trade volume positively correlates with linguistic commonality but negatively correlates with the land area of the destination country/region.

*language*_*c*_. Whether the export destination country/region shares the same language is used to measure cultural factors. The data are sourced from CEPII-GeoDist.

*relat*_*land*_*c*_. The ratio of the land area of an export destination country/region to that of China is used to measure geographical factors. The data are sourced from CEPII-GeoDist.

*contig*_*c*_. We additionally incorporate the geographical contiguity between China and the destination country (region) as a geographical factor. The data are sourced from CEPII-GeoDist.

*relat*_*rank*_*tc*_. The geographical distance between countries/regions measures the variable trade costs (34). We introduce the economic freedom index of the destination country/region into the model as a measure of fixed trade costs. Assuming that fixed trade costs have a negative impact on trade, countries/regions with higher economic freedom have lower fixed trade costs. Therefore, economic freedom is positively correlated with the export trade volume. We use the ratio of the economic freedom index of the export destination country/region to that of China in each period to measure fixed trade costs. The data are sourced from the Heritage Foundation^4^.

*fta*_*c*_. We include a dummy variable for whether a destination country/region has signed a free trade agreement (FTA) with China. The data are sourced from the Ministry of Commerce of the People's Republic of China.

### Descriptive Statistics

This paper studies the impact of the COVID-19 pandemic on Chinese exports based on the export volume of 97 categories of products exported from China to 43 major countries/regions from January 2019 to June 2020. The treatment and control groups are established by discriminating the degree of digitalization. As data on the GDP control variable of Pakistan and Burma are not available^5^, the export destinations are reduced to 40 countries/regions. The descriptive statistics of each variable are presented in Table 1.

**Table 1:** Descriptive statistics of the variables.

	**Variable**	**Obs**	**Description**	**Expected impact direction**	**Min**	**Mean**	**Max**	**Std. Dev**.
Explained variables	LnExp	71,586	China's export volume		0.00	7.26	16.55	3.40
Core explanatory Variables	CBEC	71,586	Whether it is a digital product	+	0.00	0.74	1.00	0.44
	Covid19	71,586	Whether it is after the COVID-19 outbreak	–	0.00	0.33	1.00	0.47
	CBECxCovid19	71,586	Interaction term	+	0.00	0.25	1.00	0.43
Control variables	relat_gdp	71,586	Relative economic scale of the destination country/region	+	0.00	0.11	1.81	0.24
	lndis_cap	71,586	Variable trade costs	–	6.86	8.68	9.87	0.65
	Language	71,586	Whether the same language is spoken	+	0.00	0.12	1.00	0.33
	relat_land	71,586	Relative land area of the destination country/region	–	0.00	0.19	1.78	0.37
	contig	71,586	Whether contiguous	–	0.00	0.15	1.00	0.35
	relat_rank	71,586	Fixed trade costs	+	0.83	1.18	1.54	0.17
	fta	71,586	Whether an FTA has been signed	+	0.00	0.32	1.00	0.47

## Regression Analysis

### Baseline Regression Analysis

Table 2 presents the DID estimates of the impact of the COVID-19 pandemic on Chinese exports. On the basis of Model (1), control variables are introduced one by one in Columns (1)–(4). On the basis of Model (2), Columns (5)–(8) introduce 3 types of fixed effects. Overall, the estimated coefficient of the interaction term of the DID variables indicates a significant increase in the export of digital products despite the impact of the COVID-19 pandemic. This result is in line with the assumption.

**Table 2:** Baseline regression results.

	**(1)**	**(2)**	**(3)**	**(4)**	**(5)**	**(6)**	**(7)**	**(8)**
	**LnExp**	**LnExp**	**LnExp**	**LnExp**	**LnExp**	**LnExp**	**LnExp**	**LnExp**
CBECxCovid19	0.220***	0.220***	0.220***	0.220***	0.220***	0.220***	0.220***	0.188***
	(0.061)	(0.058)	(0.058)	(0.057)	(0.057)	(0.042)	(0.042)	(0.047)
Covid19	−0.453***	−0.459***	−0.458***	−0.455***		−0.455***		
	(0.053)	(0.051)	(0.051)	(0.051)		(0.039)		
CBEC	2.650***	2.650***	2.650***	2.650***	2.650***			
	(0.035)	(0.033)	(0.033)	(0.033)	(0.033)			
relat_gdp		3.169***	2.684***	2.775***	2.808***	2.775***	2.808***	2.808***
		(0.043)	(0.047)	(0.050)	(0.050)	(0.032)	(0.032)	(0.032)
lndis_cap		−0.931***	−1.102***	−1.009***	−1.008***	−1.009***	−1.008***	−1.008***
		(0.017)	(0.020)	(0.020)	(0.020)	(0.012)	(0.012)	(0.012)
Language			0.354***	0.007	0.009	0.007	0.009	0.009
			(0.038)	(0.042)	(0.042)	(0.029)	(0.029)	(0.029)
relat_land			0.927***	0.967***	0.957***	0.967***	0.957***	0.957***
			(0.036)	(0.036)	(0.036)	(0.023)	(0.023)	(0.023)
contig			−0.561***	−0.516***	−0.513***	−0.516***	−0.513***	−0.513***
			(0.041)	(0.041)	(0.041)	(0.029)	(0.029)	(0.029)
relat_rank				0.426***	0.414***	0.426***	0.414***	0.414***
				(0.084)	(0.084)	(0.056)	(0.056)	(0.056)
fta				0.630***	0.633***	0.630***	0.633***	0.633***
				(0.028)	(0.028)	(0.018)	(0.018)	(0.018)
Month FE	No	No	No	No	Yes	No	Yes	Yes
Id FE	No	No	No	No	No	Yes	Yes	Yes
Sector*Month_FE	No	No	No	No	No	No	No	Yes
*N*	71,586	71,586	71,586	71,586	71,586	71,586	71,586	71,586
*R* ^2^	0.1242826	0.1951522	0.2021798	0.2093159	0.21702	0.649811	0.657515	0.6592744

*** *p < 0.01*.

#### Core Explanatory Variables

The estimated coefficients of *CBECxCovid*19 are all positive and significant at the 1% level, demonstrating that digital products still play a significant role in promoting Chinese exports under the pandemic impact when various factors are controlled for, leading to an increase of 18.8–22%. The estimated coefficients of *Covid*19 are all negative and significant at the 1% level, indicating that compared with the years before the pandemic, China's export of various products decreased by ~45% on average after the pandemic. The estimated coefficients of *CBEC* are all positive and significant at the 1% level, indicating that the export of digital products is higher than that of conventional products.

#### Control Variables

The baseline regression results of *relat*_*gdp* and *lndis*_*cap* are consistent with the classical gravity model assumption that trade flows are positively correlated with economic scale and negatively correlated with distance. The estimated coefficient of *language* in Column (3) is significantly positive. However, after introducing the dummy variables for economic freedom and FTAs, the coefficient is no longer significant and is very small. Speaking the same language is not significantly related to trade flows. The estimated coefficient of *contig* is still significantly negative after controlling for various factors, indicating that China's export products are more welcome in non-contiguous countries than in contiguous countries. The estimated coefficient of *relat*_*land* is significantly positive, meaning that the larger the land area of a trading partner country/region is compared to that of China or, in a sense, the better its resource endowments are compared to those of China, the higher the volume of exports from China to the country/region. Lastly, the estimated coefficient of *relat*_*rank*, namely, the trade freedom level representing variable costs, is significantly positive, indicating that the higher economic freedom of an export destination country/region compared with China means lower variable trade costs and, therefore, higher trade flows. The estimated coefficient of the binary variable, which indicates whether an export destination country/region has signed an FTA with China, is significantly positive, suggesting that the existence of an FTA reduces the market entry costs and therefore promotes the trade in imports and exports. This supports the expected direction of influence.

### Parallel Trend Test

**Figure 1:**
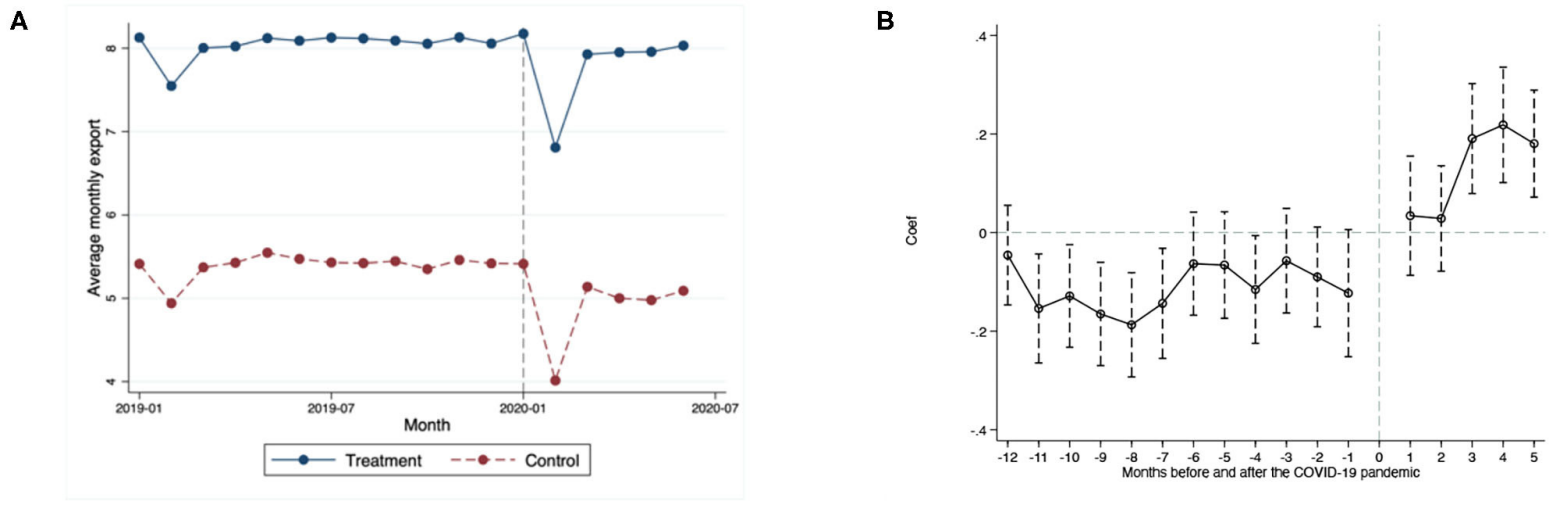
Parallel trend test. **(A)** Time series plot. **(B)** Regression coefficients of the pandemic effect.

The use of the DID approach must satisfy the parallel trend assumption (35), i.e., the exports of all products to major destination countries/regions showed the same trend before the COVID-19 pandemic. In the time series plot on the left of Figure 1, the blue continuous line and red dotted line represent the average logarithm of the monthly export volumes of the treatment group and control group, respectively. In the period before the outbreak of the pandemic, which is represented by the section to the left of the vertical dotted line for January 2020, the two groups showed largely identical trends. This finding can be preliminarily considered to be in line with the parallel trend assumption. Similar trends of the two groups are also observed in the first 3 months after the outbreak of the pandemic, meaning that both groups suffered almost the same shocks from the pandemic. However, the two groups showed different trends after the first 3 months.

The diagram on the right uses the event study method to test the parallel trends and the dynamic effects of digital trade on Chinese exports in the aftermath of COVID-19. The 0 point on the horizontal axis represents the base period of the pandemic. Each circle represents the regression coefficient of the interaction term. The vertical dotted line crossing each circle represents the 95% confidence interval of the coefficient. For the purpose of testing the parallel trends of the samples, the dummy variables for the years before the treatment are multiplied with the interaction term of the treatment variables. If the dummy variables are not significant, the parallel trend assumption is satisfied. In other words, if the regression coefficients before the pandemic are not significant, there is no significant difference between the base period and the period before the pandemic, thus supporting the parallel trend assumption. As the diagram on the right depicts, all regression coefficients of the three periods (indicated by negative figures on the horizontal axis) before the pandemic are not significant at the 5% level. Even at a relaxed significance level, no significant difference between the base period and the 6 months before the pandemic is observed, thus supporting the parallel trend assumption. When viewed dynamically, the regression coefficients of the first 2 months after the pandemic broke out are positive but not significant, which is almost in line with the time series plot. From the third month after the pandemic broke out, digital products played a significant role in promoting Chinese exports.

### Placebo Tests

#### Based on Virtual Pandemic Time Points

The influence of other unobservable factors on the explained variables may impact the conclusions of this paper. Based on the parallel trend assumption, there should be no significant difference between the export trends of the treatment group and control group before the pandemic. Therefore, if a virtual pandemic is set before January 2010, the estimated coefficients of the core variables should not be significant. Following the practices of Topalova (36), we reduce the sample to a 12-month period before the pandemic, i.e., the entire year of 2019, and set the virtual pandemic to October, November, and December 2019. Table 3 reports the regression results of the three virtual pandemic time points. As the interaction term coefficients are not significant, the direct impact of certain unobservable factors on the export trade can be ruled out.

**Table 3:** Placebo test regression results of virtual time points.

	**(1)**	**(2)**	**(3)**
	**LnExp**	**LnExp**	**LnExp**
CBECxCovid19_fake1	−0.066		
	(0.109)		
CBECxCovid19_fake2		−0.053	
		(0.076)	
CBECxCovid19_fake3			−0.025
			(0.064)
Month FE	Yes	Yes	Yes
Id FE	Yes	Yes	Yes
Sector*Month_FE	Yes	Yes	Yes
*N*	47,724	47,724	47,724
*R* ^2^	0.6554991	0.6554998	0.6554964

#### Based on Virtual Grouping

**Figure 2:**
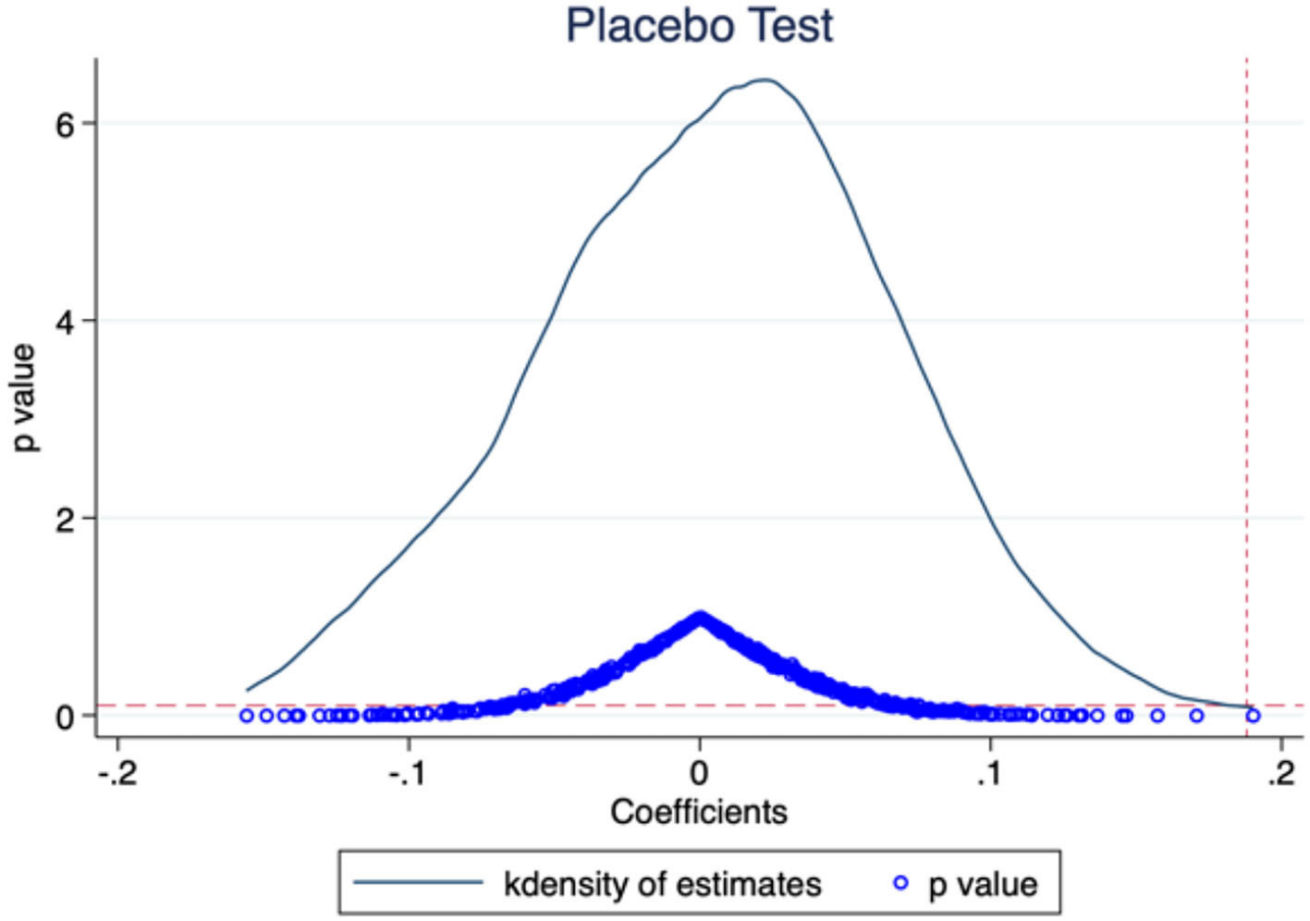
Placebo test based on virtual grouping.

It can also be questioned whether the statistical significance of the explained variables may be the result of certain random factors. The estimation bias may come from variables at the product-timing level. Therefore, we perform placebo tests by randomly grouping digital trade products (37, 38). The model consists of 97 product categories at the HS-2 level, 72 of which are included in the treatment group of digital products. We establish a virtual treatment group by randomly drawing 72 product categories and generate a virtual interaction term and add it to Model (2) for the placebo test. As the virtual treatment group is generated randomly, the interaction term in the placebo test should not have a significant influence on the dependent variable. In other words, the regression coefficient should not deviate significantly from the zero point. Meanwhile, to avoid interference by less probable events, we repeat the above process 500 times, record the interaction term coefficient and *p*-value of each regression result, and display them as kernel density plots. As shown in Figure 2, most regression coefficients are around the zero point, and the *p*-values of most estimates are over 0.1 (see the horizontal dotted line, not significant at the 10% level). The estimates of the true regression coefficients (see the vertical dotted line) are abnormal values in the placebo test. This finding indicates that our estimation results are unlikely to be obtained by chance and are therefore unlikely to be affected by other policies or random factors.

### Robustness Tests

#### Robustness Test Based on the Construction of the Control and Treatment Groups

As noted above, a product category is included in the treatment group as long as one product of that category is sold on CBEC platforms. With reference to Campello and Larrain (29), changing the methods of constructing the control and treatment groups and rerunning the Model (2) regression can help guarantee the robustness of the empirical results. Put simply, we reconstruct the groups by trichotomy to calculate the proportion of products falling under the HS-6 code segmented from each HS-2 code that are sold through CBEC channels. Then, we construct two groups of dummy CBEC variables. The first is the CBEC_low treatment group, in which over 33.3% are CBEC products, and the second is the CBEC_high treatment group, in which over 67.7% are CBEC products. In essence, we raise the criteria for discriminating digital trade products, thus reducing the number of samples in the treatment groups. Lastly, we add the two treatment groups to Model (2) for regression and obtain the results shown in Table 4.

**Table 4:** Regression results of the robustness test based on grouping.

	**(1)**	**(2)**	**(3)**
	**LnExp**	**LnExp**	**LnExp**
CBECxCovid19	0.188***		
	(0.047)		
CBECxCovid19_low		0.146***	
		(0.054)	
CBECxCovid19_high			0.122***
			(0.042)
Month FE	Yes	Yes	Yes
Id FE	Yes	Yes	Yes
Sector*Month_FE	Yes	Yes	Yes
*N*	71,586	71,586	71,586
*R* ^2^	0.6592744	0.6592137	0.6592174

*** *p < 0.01*.

We find that when the difference in the proportion of CBEC products between the treatment and control groups dwindles, the core regression coefficients decrease accordingly and are all significant at the 1% level. These results mean that the effect of the digitalization level decreases during the pandemic, which is in line with the dosage effects. Hence, the identification strategy can be considered robust.

#### Robustness Test Based on Subsamples

Considering that Chinese exports are not equally distributed among destination countries/regions, the USA, Hong Kong, and Singapore should be ruled out to avoid the effects of excessive trade volume and entrepot trade. The subsample regression results presented in Table 5 indicate a significant positive correlation, although the estimated coefficients of the core explanatory variables decrease. Interestingly, linguistic commonality is significantly positive in the subsample robustness test, whereas economic freedom shows a significant negative correlation. Clearly, the three countries/regions excluded herein have higher economic freedom than China.

**Table 5:** Regression results of the robustness test based on subsamples.

	**(1)**	**(2)**
	**LnExp**	**LnExp**
CBECxCovid19	0.188***	0.175***
	(0.047)	(0.047)
relat_gdp	2.808***	10.920***
	(0.032)	(0.090)
lndis_cap	−1.008***	−0.700***
	(0.012)	(0.013)
Language	0.009	0.158***
	(0.029)	(0.035)
relat_land	0.957***	0.834***
	(0.023)	(0.023)
contig	−0.513***	−0.777***
	(0.029)	(0.031)
relat_rank	0.414***	−0.832***
	(0.056)	(0.055)
fta	0.633***	1.018***
	(0.018)	(0.018)
Month FE	Yes	Yes
Id FE	Yes	Yes
Sector*Month_FE	Yes	Yes
*N*	71,586	66,348
*R* ^2^	0.6592744	0.6919224

*** *p < 0.01*.

### Mechanism and Heterogeneity Tests

#### Mechanism Test

In the demand-side mechanism analysis, we assume that digital trade accelerates export recovery by reducing trade costs. With reference to the explanations of trade costs in the control variable analysis, we take *relat*_*rank*_*tc*_, which stands for fixed trade costs, and *lndis*_*cap*_*c*_, which stands for variable trade costs, and make them interact with *CBEC*_i_*Covid19_*t*_, which is the original core interaction variable, to construct a new core interaction variable. For the purpose of supply-side mechanism analysis, we assume that digital trade accelerates export recovery by increasing the demand of partner countries/regions. With reference to the explanations of the economic scale of destination countries/regions in the control variable analysis, we make *relat*_*gdp*_*tc*_ interact with *CBEC*_i_*Covid19_*t*_, which is the original core interaction variable, to construct a new core interaction variable. The mechanism test focuses on the significance of the coefficient of the new core interaction terms, thus indirectly identifying the potential mechanisms through which digital trade promotes exports in the aftermath of the pandemic. The regression results of Model (3) are shown in Table 6.

**Table 6:** Regression results of the mechanism test.

	**Baseline regression**	**Demand-side mechanism**		**Supply-side mechanism**
	**LnExp**	**LnExp**	**LnExp**	**LnExp**
CBECxCovid19	0.188***			
	(0.047)			
CBECxCovid19xlndis_cap		0.025***		
		(0.005)		
CBECxCovid19xrelat_rank			0.181***	
			(0.037)	
CBECxCovid19xrelat_gdp				−0.155***
				(0.058)
lndis_cap	−1.008***	−1.014***	−1.008***	−1.007***
	(0.012)	(0.012)	(0.012)	(0.012)
relat_rank	0.414***	0.414***	0.374***	0.413***
	(0.056)	(0.056)	(0.056)	(0.056)
relat_gdp	2.808***	2.808***	2.807***	2.850***
	(0.032)	(0.032)	(0.032)	(0.036)
Month FE	Yes	Yes	Yes	Yes
Id FE	Yes	Yes	Yes	Yes
Sector*Month_FE	Yes	Yes	Yes	Yes
*N*	71,586	71,586	71,586	71,586
*R* ^2^	0.6592744	0.6593145	0.6593166	0.6592024

*** *p < 0.01*.

The supply-side mechanism regression results demonstrate that the coefficients of the new core explanatory variables are positive and significant at the 1% level. Digital technologies break through the limitation of geographical distance and increase exports mainly by reducing fixed trading costs through increased economic freedom. This means that during the pandemic period, digital trade has indeed promoted Chinese exports by reducing the trade costs on the supply side.

The demand-side mechanism regression results demonstrate that the coefficients of the new core explanatory variables are negative and significant at the 1% level. In other words, Chinese exports are negatively correlated with the economic scale of the destination country/region. This means that digital trade plays a role in promoting Chinese exports but not through economies of scale in the export destination countries/regions. This finding does not agree with our hypothetical mechanism. In the following destination country/region heterogeneity test, we will further investigate what has limited the influence of digital trade on the demand side.

#### Export Destination Country/Region Heterogeneity Tests

The heterogeneity test considers the differences in pandemic severity and economic development levels among destination countries/regions.

In terms of pandemic severity, we collect the number of confirmed COVID-19 cases in China's major destination countries/regions in the period from February to April 2020. Countries/regions with an above average number of confirmed cases are rated as high-risk areas, and the rest are rated as low-risk areas^6^. In terms of the economic development level, we divide China's major export destination countries/regions into three groups, namely, low-middle-income, middle-high-income, and high-income countries/regions, based on the World Bank's latest 2020 national income classification. Table 7 presents the results of the heterogeneity tests. In the pandemic severity heterogeneity test, the interaction regression coefficient for high-risk areas is not significant, while that for low-risk areas is positive and significant. In the income heterogeneity test, the interaction regression coefficients for all income levels are positive and significant. It can be directly observed that as the income level of a country/region decreases, the role of digital trade in promoting exports actually increases in the aftermath of the pandemic. Thus, we speculate that in the mechanism test, the demand mechanism of destination countries/regions is affected by the relatively low levels of demand of these high-income countries/regions. One possible reason is that high-income countries/regions suffer serious pandemic shocks. Therefore, we perform a pandemic risk heterogeneity test on the high-income countries/regions. The results also demonstrate that the interaction regression coefficient for high-income countries/regions in high-risk areas is not significant; however, that for high-income countries/regions in low-risk areas is significantly positive.

**Table 7:** Regression results of the country/region heterogeneity tests.

	**Risk**		**Income**			Risk*High_income	
	**Risk_high**	**Risk_low**	**High_income**	**Upper_middle**	**Lower_middle**	**HH**	**HL**
CBECxCovid19	0.107	0.210***	0.174***	0.177**	0.262***	0.119	0.191***
	(0.082)	(0.052)	(0.059)	(0.079)	(0.099)	(0.079)	(0.066)
Month FE	Yes	Yes	Yes	Yes	Yes	Yes	Yes
Id FE	Yes	Yes	Yes	Yes	Yes	Yes	Yes
Sector*Month_FE	Yes	Yes	Yes	Yes	Yes	Yes	Yes
*N*	15,714	55,872	45,396	15,714	10,476	10,476	34,920
*R* ^2^	0.8081507	0.6753944	0.6509717	0.7979662	0.8094717	0.8790593	0.6703296
Empirical *p*	0.000***		-			0.030**	

*** *p <1%*.

** *p <5%*.

When the model settings are identical among grouped samples, the regression coefficients can be compared between groups. Following Cleary (39), we test the significance of the differences in the interaction coefficients between groups after grouping regression. We obtain an empirical *p*-value based on 100 bootstrap replicates. Pandemic severity heterogeneity is significant at the 1% level. The high-income country/region heterogeneity in areas of different risk levels is significant at the 5% level. This result means that the two sets of interaction coefficients can be compared.

By further analyzing the interaction coefficients for high-risk and low-risk areas, we find that the estimated coefficients of low-risk areas are larger and more significant, implying that in the aftermath of the pandemic, digital trade has a stronger positive effect on exports to low-risk areas than on exports to high-risk areas. Higher-income countries/regions enjoy higher economic development and have higher levels of demand. In particular, we find that exports to high-income and high-risk countries/regions account for a considerable proportion of Chinese exports^7^. The non-significant estimated coefficient of high-income countries/regions severely suffering from the pandemic implies the reason why the demand mechanism fails to work.

#### Sector Heterogeneity Test

To study the sector heterogeneity of export products, we categorize the 98 product categories of the China Customs Sections and Divisions into 7 sectors and perform subsample regression on these 7 sectors. Table 8 reports the test results. The interaction regression coefficients of three sectors, namely, agricultural and food products, electromechanical instruments and vehicles, and garments, shoes and hats, are positive and significant at the 1% level, while those of other sectors are not significant. This result means that digital trade has mainly promoted the growth in exports of these 3 sectors after the outbreak of the COVID-19 pandemic.

**Table 8:** Regression results of the sector heterogeneity test.

	**Full sample**	**Agricultural and food products**	**Electromechanical instruments and vehicles**	**Chemicals, minerals and metals**	**Garments, shoes and hats**	**Rubber and leather**	**Clocks, watches and toys**	**Woods, paper and non-metals**
CBECxCovid19	0.188***	0.448***	0.386***	0.079	0.316***	−0.314	0.000	−0.096
	(0.047)	(0.123)	(0.148)	(0.076)	(0.085)	(0.240)	(.)	(0.138)
Month FE	Yes	Yes	Yes	Yes	Yes	Yes	Yes	Yes
Id FE	Yes	Yes	Yes	Yes	Yes	Yes	Yes	Yes
Sector*Month_FE	Yes	Yes	Yes	Yes	Yes	Yes	Yes	Yes
*N*	71,586	17,712	7,380	18,450	13,284	3,690	3,690	6,642
*R* ^2^	0.6592744	0.5005582	0.7587776	0.6696178	0.5428716	0.731179	0.7764769	0.7709698

*** *p <1%*.

We further explain sector heterogeneity from the demand perspective. The COVID-19 shocks to demand evidently differ between goods that are necessary and unnecessary for pandemic prevention. Digital trade further enhances this difference. For example, “panic buying” resulting from physical distance has dramatically increased the demand for agricultural and food products, face masks, and other pandemic prevention necessities. As COVID-19 spreads quickly around the world, the demand for medical instruments such as ventilators and medical textiles such as protective garments increases sharply. While helping recover the supply, digital trade accelerates the export recovery of these three sectors. In contrast, digital trade has no significant effect on mitigating the negative COVID-19 shocks to the demand for goods that are unnecessary for pandemic prevention, such as chemicals, minerals and metals, rubber and leather, clocks, watches and toy, and woods, paper and non-metals.

## Conclusion and Discussion

### Conclusion

We base our study on monthly export data on 97 product categories under the China Customs Section and Division system that China exports to 40 major countries/regions in the period from January 2019 to June 2020. Using the DID method, we empirically study how digital trade has influenced Chinese exports under the natural shocks of COVID-19 and discuss the effects of digital trade on China's exports in the aftermath of the pandemic and the potential mechanisms.

Through empirical analyses, we obtain a number of findings.

First, the overall regression demonstrates that digital trade is significantly and positively correlated with Chinese exports in the pandemic context, suggesting that digital trade has positively promoted Chinese exports. There might be two reasons for this result. The first is the technical advantages of China. China ranks among the top countries worldwide in the use of the Internet, wireless broadband, and mobile terminals, and it has submitted nearly 20% of the world's patent applications in the fields of big data, cloud computing and artificial intelligence. Meanwhile, China ranks first in blockchain-related patent applications, filing ~33,000 such patent applications. Social media and search engines are also in high demand overseas. The global service capability of China's BeiDou Navigation Satellite System is evidently enhanced. The second reason is the institutional advantages represented by the number of digital trade demonstration zones, 12 digital service export bases, 29 cultural export bases, 28 service trade innovation and development pilot areas, and 31 service outsourcing demonstration cities. Additionally, we found two control variables of interest. The first one *language* is not significantly related to trade flows. One possible reason may be that the use of machine translation mitigates the negative impact of language barriers on trade. Brynjolfsson et al. (40) found that the machine translation provided on CBEC platforms can boost growth in trade. The second one *relat*_*land* in the estimated coefficient of is significantly positive and the result differs from our expectations. One possible reason is that these countries/regions are facing more serious shocks under the pandemic and are therefore resorting to more imports.

Second, the sudden outbreak of COVID-19 has significantly inhibited Chinese exports overall. The dynamic parallel trend test demonstrates that digital trade started to significantly promote exports from the third month after the outbreak of the pandemic, implying that the lockdown had a negative effect on export trade but that digital trade quickly recovered after the lockdown was lifted.

Third, the mechanism test indicates that in the case of China, digital trade accelerates export recovery and growth mainly through the supply mechanism rather than the demand mechanism. One potential reason may be that the samples are taken from a relatively short time series after the pandemic. At least as viewed from the situation with a short period after the pandemic, digital trade did not play a role in increasing the demand of partner countries/regions.

Fourth, the country/region heterogeneity test shows that digital trade positively promotes exports to low-risk areas more strongly than it promotes those to high-risk areas, even in high-income countries/regions, implying that the pandemic control effect in destination countries/regions can impact the promoting effect of digital trade and that high-income and high-risk countries/regions may have potentially affected the effect of the demand mechanism.

Fifth, the sector heterogeneity test indicates that digital trade has significantly promoted the export of goods that are necessary for pandemic prevention.

Finally, a series of robustness tests, including the parallel trend test and placebo test, prove that our conclusions are robust.

### Implications for Health Economics

The impact of COVID-19 will foreseeably continue for a period of time, and digital trade will play a more important role in global trade in the post-pandemic era. More importantly, reflecting on events such as COVID-19 that have had an enormous impact on economic growth is conducive to promoting sustained and healthy economic development. Taking into account digital technology in response to major health emergencies, through the storage, matching, analysis and visualization of large data on population movements, this study reveals the relevance of human behavior and processes, and it provides development opportunities for health economics. We propose the following implications for health economics from the perspective of digital technology:

1. Global health management cooperation. At present, countries around the world have not yet reached a consensus on health management measures such as epidemic control and vaccination. Our heterogeneity test results point out that differences in health management have led to an imbalance between demand and supply; as a result, digital technology has been unable to maximize trade and economic recovery. Therefore, we suggest that global health organizations must unite, pay attention to the impact of the pandemic on interpersonal relations and economic recovery, increase investment in human resources such as health, and accelerate the integration of digital technology and the healthcare industry.
2. Establishing an early warning and emergency response mechanism for public health crises. It is believed that COVID-19 will continue to exist for a period of time in the future. Therefore, the post-COVID-19 period is particularly important for retrospective data in epidemic-related or health fields. From the perspective of this article, it is necessary to focus on and address the labor factor in the supply chain (demand and supply). The digitalization of the relationship between supply and demand is an important factor in the supply chain construction (41). Health is a human resource, and early warning and emergency mechanisms need to be verified. Therefore, we call for establishing a national-level health strategy and social care system, flexibly implementing social distancing and epidemic investigations, giving full play to the role of the government as an information intermediary (42, 43), and providing sufficient resources to support economic recovery.
3. Establishing medical and health big data. Health economics research also uses the DID model to analyze the sample data and to analyze and explain the value of health. Pandemic-related data showed an exponential growth trend during COVID-19. These open health and medical data make the data collection and analysis of health economics possible. We suggest using health big data to pay attention to the impact of personal ideas and social norms as well as other cultural factors on health behaviors. Personal health behaviors include the willingness to receive the COVID-19 vaccine, the willingness to wear masks, the behavior of people gathering together, and potential challenges to the fairness and accessibility of the national medical security system.

### Limitations and Future Research

First, this paper focuses only on China, where an enormous number of confirmed cases of COVID-19 occurred in the early stage of the pandemic, to study the effect of digital trade on Chinese exports, but research can go further to cover more objects of study. China resolutely enforced a series of strict control measures such as stopping production and work soon after the outbreak of the pandemic to curb its spread despite the sacrifice of economic effects. In this respect, globally, China represents a rare case. The objects of future research may expand to include high-risk countries in the post-pandemic era and countries where digital trade is well developed. By incorporating the contribution of digital trade in those countries after the outbreak of the pandemic, the conclusions of this paper can be more solid and robust.

Second, the time series of the study cover only the 6 months after the outbreak of the pandemic. This decision was made to focus on how digital technologies have promoted Chinese exports in the short term. Furthermore, it is due to data limitations caused by the changes in China Customs' statistical coverage in July 2020. Short-term time series actually pose challenges for variables selection. Many indicators that reflect national capabilities, such as digital capabilities, and some lagging variables, such as FDI (44), are not exhaustively listed in the empirical regression, but are controlled with fixed effects. Similarly, we did not find appropriate short-term instrumental variables to control for endogeneity problems caused by potential reverse causality. Based on the regular pandemic situation of COVID-19, it is particularly important to investigate the mechanism through which digital technologies have a lasting effect on global trade. In future research, it is necessary to obtain data from a longer time series and thus explore the action mechanism of digital trade in more detail.

## Data Availability Statement

The original contributions presented in the study are included in the article/supplementary material, further inquiries can be directed to the corresponding author.

## Author Contributions

All authors undertook research, writing, review tasks throughout this study, read, and agreed to the published version of the manuscript.

## Funding

This work was supported by the Major Program of the National Social Science Foundation of China (Grant Number 20&ZD124), the National Social Science Foundation of China (Grant Numbers 21CJY024, 20BJL040, 19BJL108, 19BJL185, and 20BJY110), the National Natural Science Foundation of China (Grant Numbers 71773115, 72174180, 72074195, 71973129, 72072162, 72173073, 71503003, and 72174112), the Philosophy and Social Science Program of Zhejiang (Grant Numbers 22NDQN290YB, 22QNYC13ZD, and 21NDYD097Z), and the Humanity and Social Science Foundation of Ministry of Education of China (Grant Numbers 21YJA790043, 21YJA630037, 19YJA790107, 19YJA630092, 18YJA790088, and 21YJCZH213).

## Conflict of Interest

XL was employed by the company China State Construction International Investments (Zhejiang Province) Ltd., Hangzhou, China. ZDu was employed by the company Jinhua JG Tools Manufacturing Co., Ltd., Jinhua, China. ZDo was employed by the company Sales Department, Estone SRL, Carrara, Italy. The remaining authors declare that the research was conducted in the absence of any commercial or financial relationships that could be construed as a potential conflict of interest.

## Publisher's Note

All claims expressed in this article are solely those of the authors and do not necessarily represent those of their affiliated organizations, or those of the publisher, the editors and the reviewers. Any product that may be evaluated in this article, or claim that may be made by its manufacturer, is not guaranteed or endorsed by the publisher.

## Footnotes

^1^Guidance by General Office of the State Council on the Promotion of Cross-Border E-Commerce in a Healthy and Rapid Way. Available online at: http://www.gov.cn/zhengce/content/2015-06/20/content_9955.htm (accessed on 20 June 2016). (In Chinese).

^2^China Customs' Sections and Divisions of export products are in accordance with the hierarchy and coding order under the *Commodity Classification for China Customs Statistics*, which is compiled on basis of the Harmonized Commodity Description and Coding System (HS).

^3^In 1992, the commodity coding system was changed to an 8-digit system based on the Harmonized Commodity Description and Coding System (HS). The first 6 digits form the HS code, and the last 2 digits form a subcatalog to serve the needs of China's customs duty, statistics and trade administration.

^4^The economic freedom index is a total score of 12 components, such as government expenditure, the tax burden, business freedom, labor freedom, trade freedom, and financial freedom. It is divided into five levels: complete freedom, relative freedom, limited freedom, relatively suppressed, and suppressed.

^5^The CEIC database https://info.ceicdata.com does not provide the monthly nominal GDP data of Pakistan and Burma. We also tried to obtain such data from the International Monetary Fund (IMF), the statistics of the Association of Southeast Asian Nations (ASEAN), and the Central Statistical Organization of Burma but failed.

^6^In accordance with the COVID-19 Data Repository of the Center for Systems Science and Engineering (CSSE) at Johns Hopkins University, among the major export destination countries/regions studied in this paper, the USA, Spain, Italy, the UK, Germany, France, Turkey, Iran, and Brazil (ranked from high to low by the number of infections) are classified as high-risk areas.

^7^The percentage of exports to high-income and high-risk countries/regions total 31.28% of China's export volume, including 19.98% to the USA, 3.83% to Germany, 3.10% to the UK, 1.60% to France, 1.52% to Italy and 1.25% to Spain, based on the sample calculation.
